# Draft Genome Sequence of *Pantoea sp.* Strain MHSD4, a Bacterial Endophyte With Bioremediation Potential

**DOI:** 10.1177/11769343231217908

**Published:** 2024-03-13

**Authors:** Dimpho Michelle Morobane, Khuthadzo Tshishonga, Mahloro Hope Serepa-Dlamini

**Affiliations:** Department of Biotechnology and Food Technology, Faculty of Science, University of Johannesburg, Doornfontein Campus, Doornfontein, Johannesburg, South Africa

**Keywords:** *Pantoea*, whole genome sequencing, bioremediation, bacterial endophyte

## Abstract

*Pantoea* sp. strain MHSD4 is a bacterial endophyte isolated from the leaves of the medicinal plant *Pellaea calomelanos.* Here, we report on strain MHSD4 draft whole genome sequence and annotation. The draft genome size of *Pantoea* sp. strain MHSD4 is 4 647 677 bp with a G+C content of 54.2% and 41 contigs. The National Center for Biotechnology Information Prokaryotic Genome Annotation Pipeline tool predicted a total of 4395 genes inclusive of 4235 protein-coding genes, 87 total RNA genes, 14 non-coding (nc) RNAs and 70 tRNAs, and 73 pseudogenes. Biosynthesis pathways for naphthalene and anthracene degradation were identified. Putative genes involved in bioremediation such as *copA, copD, cueO, cueR, glnGm*, and *trxC* were identified. Putative genes involved in copper homeostasis and tolerance were identified which may suggest that *Pantoea* sp. strain MHSD4 has biotechnological potential for bioremediation of heavy metals.

## Introduction

Endophytes are microorganisms such as bacteria and fungi, that colonize and inhabit plant tissues for all or part of their life cycle without causing harm or damage to the host plant. This community of microbes forms beneficial relationship with their plant host counterpart and exhibit plant growth and development properties through the production of unique and various biochemical compounds.^1,2^ Of interest in this study are bacterial endophytes, which like fungal endophytes are known to provide nitrogen-fixation benefits to the plant and may secrete secondary metabolites and siderophores, which allow for pathogenic resistance and absorption of iron in host plants.^3^ Furthermore, the interactions between endophytic bacteria and plants promote plant’s ability to withstand extreme and unfavorable environmental conditions.^4^

*Pantoea* species are widely distributed in nature and have been isolated from various ecological habitats such as soil, water, animals, the human gut and plants.^5^ The genus *Pantoea* belongs to the family Erwiniaceae and is composed of over 20 recognized species with the genus type species as *Pantoea agglomerans*.^6^ The members of *Pantoea* are Gram-negative, facultative anaerobic, rod-shaped, commonly motile, and are yellow-pigmented due to the production of carotenoids.^7,8^ Endophytic *Pantoea* species produce antimicrobials such as pantocin A and B as well as D-alanylgriseoluteic acid (AGA) and may possess bioremediation potential.^6,9^

Bioremediation is a cleaning-up technique in polluted environments by transforming enzymes during the biosorption of different toxic metals.^10,11^ The environmental pollution due to various toxic metals is a serious concern in the food sector because of decreasing crop yields and food quality due to the overuse of fertilizer, mulching, and agricultural pesticides which cause heavy metal pollution of soils.^12^ Bioremediation has been identified as a good technique for controlling pollution and treatment of toxins. Several studies have shown that endophytes can be used in bioremediation techniques because of their ability to improve plant response to a range of stressors.^13^ Endophytes improve the ability of plants to survive in various environmental conditions such as drought, nitrogen deficiency, pathogens, and salinity, and such impact has a very important contribution to the bioremediation of heavy metals.^14^

Whole genome sequence analysis of bacterial endophytes is a valuable tool that is necessary for the identification of genes that are responsible for key endophytic biochemical activities such as nitrogen fixation, nutrient acquisition, and phytohormone production. Individual genome sequences improve the study of plant-microbe interactions as well as data analysis in requisite endophytic metagenomics, proteomics, and transcriptomics studies.^15,16^ Furthermore, obtained whole genomic sequence data is more useful to advance taxonomical resolution of bacterial species in contrast to more traditional methods such as 16S rRNA gene sequencing analysis.^17^ The objective of this study was to sequence, assemble, and annotate the genome of the endophytic bacterium *Pantoea* sp. strain MHSD4 previously isolated from medicinal plant *Pellaea calomelanos.*^18^ Previous unpublished data indicated that strain MHSD4 could degrade tannic acid, thus we hypothesized that the strain has bioremediation capabilities, which could be additionally investigated through *in silico* analysis.

## Materials and Methods

### Bacterial sample preparation and genomic DNA extraction

The endophyte, *Pantoea* sp. strain MHSD4 was previously isolated and identified in a study by Mahlangu and Serepa-Dlamini,^18^ and the 35% glycerol stock cultures of the endophyte were preserved at −80°C. In this study, the endophyte was re-cultured on tryptic soy agar (TSA) for 48 hours at 28°C.^19^ The total genomic DNA of the bacterial culture was extracted from pure colonies using the Nucleospin^®^ Microbial DNA extraction kit (Macherey-Nagel, Germany) according to the manufacturer’s protocol. The extracted DNA quality and quantity was determined using the Implen Nanophotometer N60 (Implen GmbH, Germany).

### Genome sequencing, de novo assembly and annotation

The extracted DNA was sent for whole genome sequencing at the Agriculture Research Council (ARC) in Onderstepoort, South Africa, a commercial service provider. The Illumina HiSeq platform was used, and pair-ended libraries (2 × 150 bp) were created with NextEra DNA library preparation kit. A total of 781 234 paired-end reads at 25× coverage were obtained from this workflow. Pre-annotation analyses were done on the Galaxy web platform (https://usegalaxy.org) using default parameters.^20^ The quality of the raw reads obtained was assessed with FastQC v 0.72. The *de novo* assembly was conducted with Unicycler v 0.4.6.0 followed by the assembly quality assessment with QUAST v 0.4.6.3 both using default settings.^21,22^ Annotation of the draft genome was done using National Center for Biotechnology Information Prokaryotic Genome Annotation Pipeline (NCBI-PGAP).^23^ The genome was also uploaded and annotated on rapid annotations using subsystems technology (RAST) server.^24^

### Phylogenomic analyses

The genomic data was uploaded on Type Strain Genome Server (TYGS) (https://tygs.dsmz.de) for whole genome based taxonomic comparison with other published type strains.^25^ The average nucleotide identity (ANI) value was determined on the Orthologous Average Nucleotide Identity Tool (OAT) v 0.93.10.^26^ Islandviewer 4 Web (https://www.pathogenomics.sfu.ca/islandviewer) was used to identify the genomic islands (GI) by screening the PGAP annotation file obtained from NCBI.^27^ The shared and unique genes clusters of strain MHSD4 were determined by comparing it to other closely related species using the EDGAR 2.0 platform.^28^

The whole genome shotgun project data has been deposited at DDBJ/ENA/GenBank with BioProject number PRJNA783158 and BioSample number SAMN23419518 under the accession number JAJNDH000000000. The version presented here is JAJNDH010000000.

## Interprepation of Data Set

The assembly of the draft genome produced 41 contigs with N_50_ value of 550 575 base pairs (bp). The genome size obtained was 4 647 677 bp and the G+C content was 54.2%. The average genome sizes and G+C content of *Pantoea* species range from 4.6 to 6.3 Mb and 52% to 55%, respectively.^6^ This is consistent with *Pantoea* sp. strain MHSD4. The total number of genes of MHSD4 predicted were 4395 which included 4235 protein-coding genes. The number of RNA genes were 87, 14 non-coding (nc) RNAs and 70 tRNAs, and 73 pseudogenes were also present. The genome attributes of *Pantoea* sp. strain MHSD4 are listed in Table 1.

**Table 1.** Annotated genome statistics of *Pantoea* sp. strain MHSD4.

Genomic attributes	Value
Number of contigs	41
Size (bp)	4 647 677
G+C content (%)	54.2
Contig N_50_	550 575
Total number of genes	4395
Protein coding genes	4235
Total RNAs	87
ncRNAs	14
tRNAs	70
Pseudogenes	73

Abbreviations: bp, base pairs; ncRNA, non-coding RNA, tRNA, transfer RNA.

**Figure 1.**
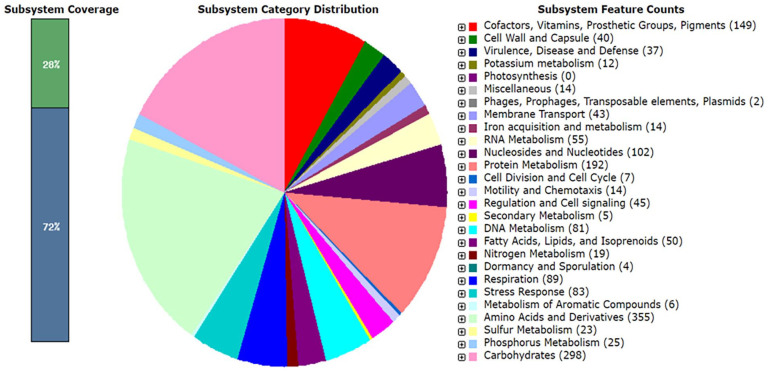
The subsystem distribution of *Pantoea* sp. strain MHSD4 genome based on RAST annotation server.

**Figure 2.**
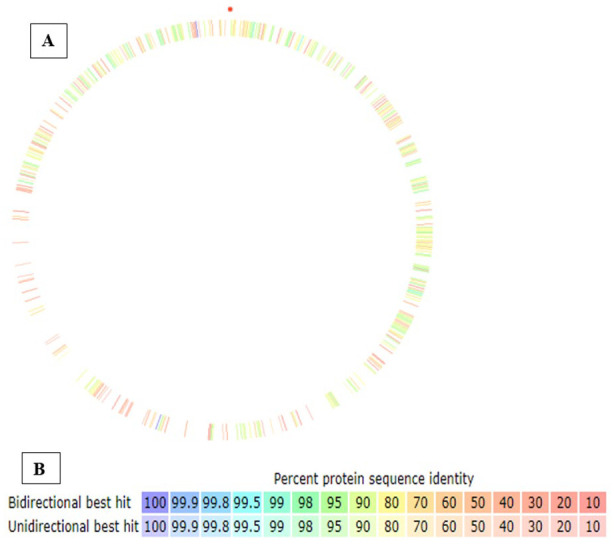
(A) *Pantoea* sp. strain MHSD4 genome compared to *Pantoea stewartii* DC283 as a reference genome using RAST sequence-based comparative tool. (B) Percentage color coordination of genome comparison. Bidirectional best hit indicates both forward and reverse hits.

Phylogenomic classification of the draft genome *Pantoea* sp. strain MHSD4 was performed using the Type Strain Genome Server for whole genome based taxonomic analysis. The Genome-based taxonomic analysis indicates that MHSD4 was closely related to *Pantoea hericii* JZB 2120024^T^ as shown in Figures 1 and 2 (Supplemental Data).

Based on 16S rDNA sequences, strain MHSD4 displayed a close relation to *Pantoea hericii* JZB 2120024^T^ and formed a polyphyletic relationship with *Pantoea eucalypti* LMG 24197 Figure 2 (Supplemental Data). A multilocus sequence analysis (MLSA) study revealed that *P. hericii* JZB 2120024^T^ which was isolated from fruiting bodies of edible mushrooms, was close phylogenetic relative with *P. eucalypti* DSM 23077^T^.^29^ Additionally, based on 16S rRNA gene sequence analysis, the novel isolate was closely related to *P. eucalypti* LMG 24197 which supports the findings shown in Figure 1 (Supplemental Data).^29^

Moreover, Figure 2 (Supplemental Data) shows that based on whole genome sequence analysis, strain MHSD4 was closely related to *P. eucalypti* LMG 24197 and both strains had a polyphyletic relationship with *P. hericii* JZB 2120024^T^. *Pantoea eucalypti* LMG 24197 was isolated from the leaves of eucalyptus plant which has many medicinal benefits.^30^ This elucidates that *Pantoea* strains are widely distributed in the environment and may also be associated with numerous medicinal plants.

Average nucleotide identity (ANI) and digital DNA-DNA hybridization (dDDH) values are both popular methods of discriminating between bacterial taxons and are imperative tools for genomic studies.^31,32^ The dDDH values calculated with 3 different formulas between *Pantoea* sp. MHSD4 and *P. hericii* JZB 2120024^T^ were 81.3%, 93.9%, and 86.3%, respectively, with a G+C content difference of .44% (Table 1, Supplemental Data). Moreover, the dDDH values between strain MHSD4 and *P. eucalypti* LMG 24197 were 95%, 93.8%, and 96.6%, respectively, and a G+C content difference of .11%. The dDDH values exceeded the threshold of >70% as a criterion to determine the similarity between bacterial strains as well as for the delineation of bacterial species.^33^ Furthermore, the lesser G+C content difference between strain MHSD4 and *P. eucalypti* LMG 24197 may be an indicator that the 2 species are phylogenetically closer in comparison to other type strains.^34^

RAST identified a total of 1490 subsystem feature counts (Figure 1) with amino acids and derivatives subsystem category dominant with 23.8% followed by carbohydrate metabolism genes with 20%. The least number of genes identified on the genome were only 2 belonging to the phages, prophages, transposable elements, and plasmids subsystem category. Genes identified within the genome of strain MHSD4 that supported the endophytic lifestyle included siderophore production as well as other iron acquisition and metabolism genes, nitrogen, phosphorus, and potassium metabolism genes.^21^ Furthermore, virulence, disease, and defense, phytohormone production, and stress tolerance genes play a significant role in the host plant growth promotion and development and the symbiotic association between bacterial endophytes and plants.^10,35^

The RAST sequence-based comparative tool was used to compare the genomes of strain MHSD4 to *Pantoea stewartii* DC283 as a reference strain as shown in Figure 2A and B. There were genome gaps observed (Figure 2A), however there was a protein sequence similarity range of 60% to 99.5% for both forward and reverse hits within the 2 compared strains. The genome gaps may substantiate some gene deletions within the genome of strain MHSD4.^36^

**Figure 3.**
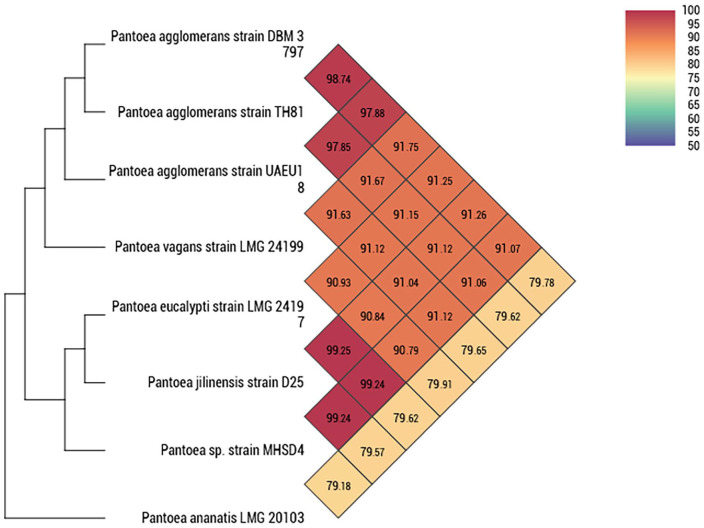
Heatmap generated with OAT software indicating OrthoANI values of *Pantoea* sp. strain MHSD4 and closely related *Pantoea* species.

Figure 3 shows the different OrthoANI values between various *Pantoea* genome species. The calculated OrthoANI values exhibited between strain MHSD4 and *P. eucalypti* LMG 24197 was 99.24%. This value was also the same between strain MHSD4 and *P. jilinensis* strain D25. The OrthoANI threshold range is approximately 95% to 96%, which was exceeded by both findings.^26^ This indicates high similarity between *Pantoea* sp. MHSD4 and *P. eucalypti* LMG 24197 and *P. jilinensis* D25, respectively. The dDDH and ANI values were congruent indicating that strain MSHD4 is closely related to *P. eucalypti* LMG 24197. In a previous study, *P. jilinensis* D25 was found to be an effective yet novel biocontrol agent against the crop disease, gray mold.^37^ This may suggest that *Pantoea* sp. strain MHSD4 may have valuable and diverse applications given the qualities of its related species.

**Figure 4.**
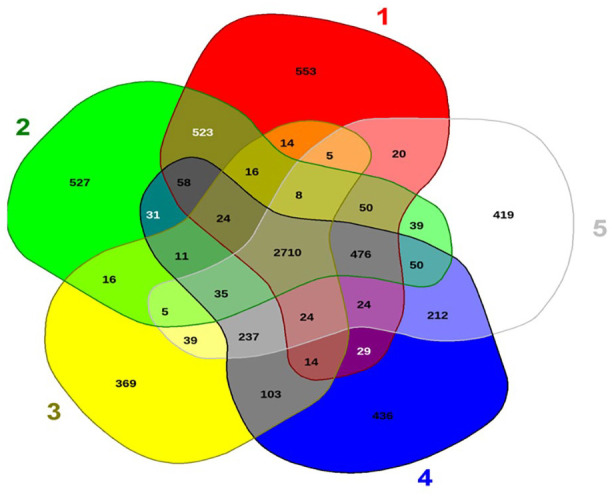
Venn diagram of the unique and shared genes of *Pantoea* sp. strain MHSD4 and selected comparison *Pantoea* species, 1. *Pantoea allii* strain LMG24248; 2. *Pantoea ananatis* LMG2665; 3. Pantoea conspicua strain LMG 24534; 4. *Pantoea pleuroti* strain JZB 2120015; 5. *Pantoe sp*. Strain MHSD4.

**Figure 5.**
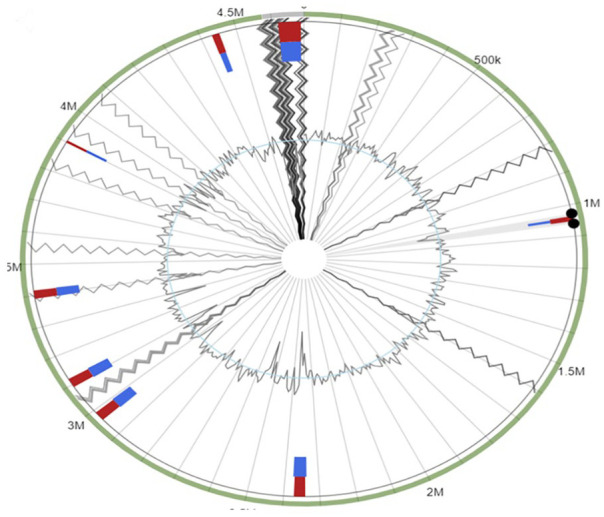
Genomic islands genes within *Pantoea* sp. strain MHSD4 aligned against reference genome within *Pantoea ananatis* LMG 20103, complete genome. Islandviewer 4 predicted a total of 8 genetic islands. The genomic islands genes prediction methods are represented by the following colors: IslandPath-DIMOB (blue), integrated detection (red). Contig boundaries are indicated by the gray lines.

*Pantoea* sp. strain MHSD4 shared 35 common genes with other closely related *Pantoea* species and only shared 5 genes with *Pantoea ananatis* LMG2665 (Figure 4). Common genes among *Pantoea* sp. strain MHSD4 and all selected comparison *Pantoea* species were found to be a total of 2710 (Figure 4). *Pantoea* sp. strain MHSD4 consists of 419 unique genes that encode transport proteins and transcriptional regulators which are important genes for bacterial endophytes that were previously identified in other bacterial species.^38,39^ Several sets of genes acquired through horizontal gene transfer were found in the genome of *Pantoea* sp. strain MHSD4 (Table 2, Supplemental Data). A total of 8 GI (Figure 5) were identified in *Pantoea* sp. strain MHSD4 genome when aligned to a reference genome *Pantoea ananatis* LMG 20103. The genomic islands’ genes clustered details are shown in Table 2 (Supplemental Data).

Biosynthesis pathways of phytohormones of *Pantoea* sp. strain MHSD4 were determined and found to consist of various essential plant growth promoting hormones such as gibberellins, auxin, abscisic acid, salicylic acid, jasmonic acid, ethylene and cytokinin (Figure 3, Supplemental Data). *Pantoea* sp. strain MHSD4 has shown the ability to degrade naphthalene and anthracene (Figure 3, Supplemental Data), which indicates a critical role in bioremediation as anthracene and naphthalene support the growth of microorganisms (Table 2).^40^

**Table 2.** Genes responsible for Bioremediation that were identified from *Pantoea* sp. strain MHSD4 genome.

Genes	Product	Function
*copA*,	Copper-exporting P-type ATPase CopA	Exports Cu^+^ from the cytoplasm to the periplasm and Binds 2 Cu^+^ ions per monomer, which are transferred to periplasmic copper chaperone CusF upon ATP hydrolysis.^41-44^
*copD*	Copper homeostasis membrane protein *CopD*	Involved in the uptake of copper to the cytoplasm.^45^
*cueO*	Multicopper oxidase CueO	involved in copper tolerance under aerobic conditions.^46^
*cueR*	Cu(I)-responsive transcriptional regulator	activates transcription of the copper tolerance genes copA and cueO^41,42^
glnG	Nitrogen regulation protein NR(I)	controls expression of the nitrogen-regulated (ntr) genes in response to nitrogen limitation.^47^
trxC	Thioredoxin TrxC	Performs a major role in surviving under redox stress, generated by the host.^48^
arsC	Arsenate reductase (glutaredoxin)	Involved in resistance to arsenate. It is arsenate reductase enzymes, that transform arsenate to arsenite prior to the extrusion of the latter oxyanion.^49^
tusA	Sulfurtransferase TusA	Function as a sulfurtransferase in tRNA modification and molybdenum cofactor biosynthesis.^50^
bfd	Bacterioferritin-associated ferredoxin	Is an iron storage and detoxification protein that differs from other ferritins by its ability to bind heme cofactors.^51^
soxS	Superoxide response transcriptional regulator	reduce the oxidative stress generated by metal-induced reactive oxygen species (ROS)^52^

In this study, *Pantoea* sp. strain MHSD4 genome specifically showed a presence of copper resistance genes such as *copA, copB and copR* which code respectively for a copper-translocating P-type ATPase, copper resistance protein B, and transcriptional activator protein.^53,54^ A few studies have identified copper-resistant gene clusters and operons in different bacterial species such as *Pseudomonas* and *Xanthomonas.*^55,56^ These genes are induced in varying levels of copper (Cu) ions and attribute to the mechanisms of copper ion transport across bacterial cells and the oxidation of Cu^+^ to less toxic Cu^2+^ ions.^55,56^ Additionally, another significant gene identified, coded for a type of multi-copper oxidase which is also known to play a role in the copper homeostasis of bacterium thus increasing stress tolerance against the heavy metal.^57^ Multicopper oxidase encoded by *cueO* which is involved in copper tolerance under aerobic conditions has been identified. The putative multi-copper oxidase has previously been reported to confer copper tolerance in *Escherichia coli.*^58^ The presence of this gene in the periplasm protected alkaline phosphatase from copper-induced damage. The multi-copper oxidase has been implicated in possession of an extensive methionine-rich region proximal to the copper centers that is reported to be involved in the binding of copper .^58^

The presence of these genes may indicate biotechnological potential for this endophytic strain in terms of conferring resistance to host plants or as a biological key player for copper remediation in the environment.^14^

High throughput sequencing of bacterial genomes allows for the identification and functional classification of genes within endophytic bacteria. This study reports on the draft genome of the bacterial endophyte, *Pantoea* sp. strain MHSD4 which was previously isolated from the leaves of the medicinal plant *Pellaea calomelanos*. Biosynthesis pathways of phytohormones consisting of various essential plant growth-promoting hormones were identified which are significant in supporting the endophytic lifestyle of the bacterium. Further comparative genomic studies will be imperative in providing valuable insights on members of the genus *Pantoea* as well as bacterial endophyte diversity and their interactions with plant hosts.

## Supplemental Material

sj-docx-1-evb-10.1177_11769343231217908 – Supplemental material for Draft Genome Sequence of Pantoea sp. Strain MHSD4, a Bacterial Endophyte With Bioremediation Potential Supplemental material, sj-docx-1-evb-10.1177_11769343231217908 for Draft Genome Sequence of Pantoea sp. Strain MHSD4, a Bacterial Endophyte With Bioremediation Potential by Dimpho Michelle Morobane, Khuthadzo Tshishonga and Mahloro Hope Serepa-Dlamini in Evolutionary Bioinformatics
