# Supplementary material for: Draft Genome Sequence of Pantoea sp. Strain MHSD4, a Bacterial Endophyte With Bioremediation Potential
Source: Evol Bioinform Online. 2024 Mar 13;20:11769343231217908. doi: 10.1177/11769343231217908 (PMC10938601; doi:10.1177/11769343231217908)
Supplement: sj-docx-1-evb-10.1177_11769343231217908 – Supplemental material for Draft Genome Sequence of Pantoea sp. Strain MHSD4, a Bacterial Endophyte With Bioremediation Potential [file sj-docx-1-evb-10.1177_11769343231217908.docx]

**Draft Genome Sequence of *Pantoea sp.* strain MHSD4, a bacterial endophyte with bioremediation potential**

**Dimpho Michelle Morobane, Khuthadzo Tshishonga, and Mahloro Hope Serepa-Dlamini**

Department of Biotechnology and Food Technology, Faculty of Science, University of Johannesburg, Doornfontein Campus, South Africa


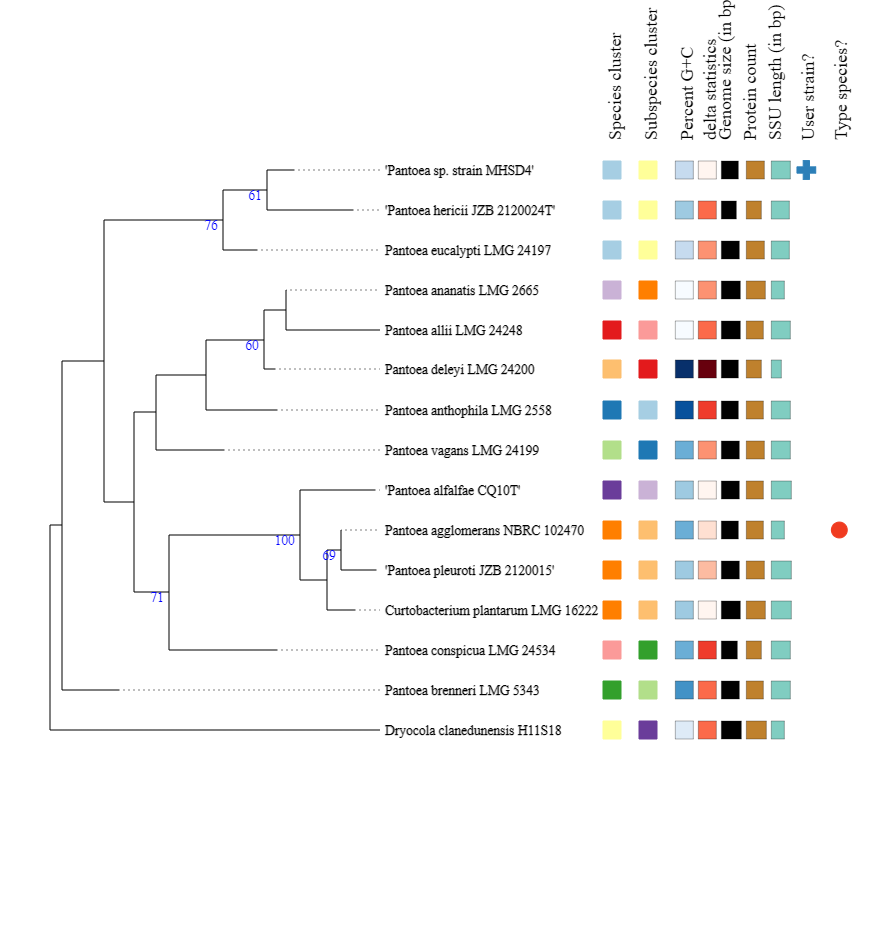


Fig. 1: Tree inferred with FastME 2.1.6.1 from GBDP distances calculated from 16S rDNA gene sequences. The branch lengths are scaled in terms of GBDP distance formula *d_5_*. The numbers above branches are GBDP pseudo-bootstrap support values > 60% from 100 replications, with an average branch support of 58.2 %. The tree was rooted at the midpoint.


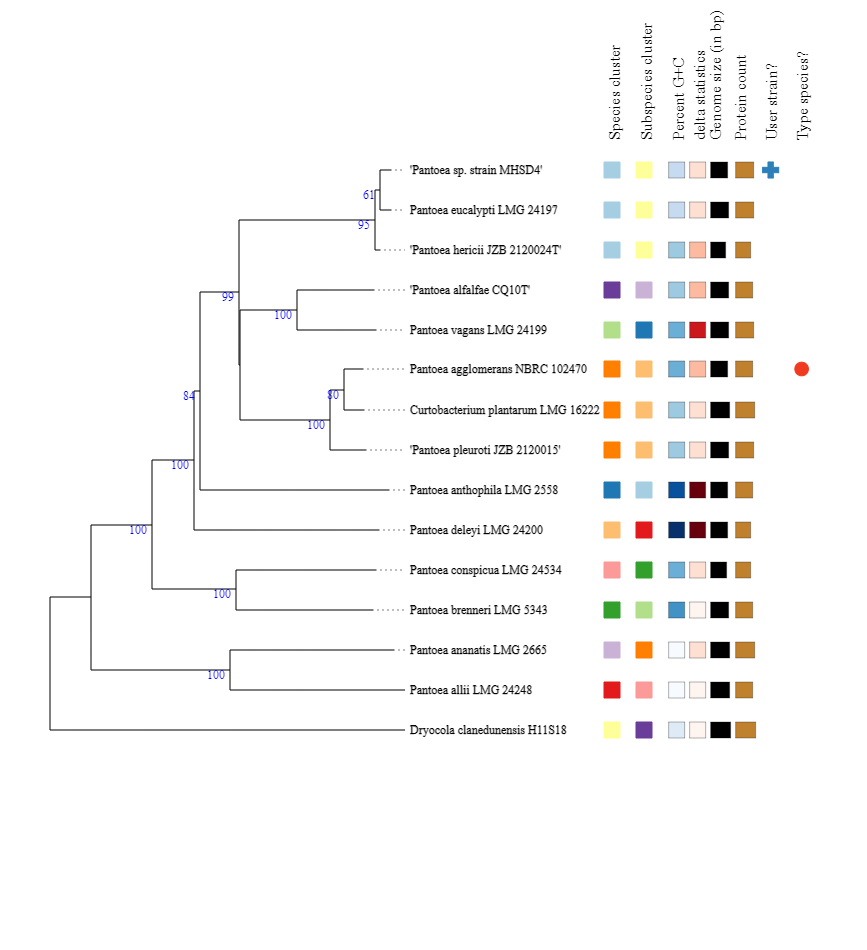


Fig. 2: Tree inferred with FastME 2.1.6.1 from GBDP distances calculated from genome sequences. The branch lengths are scaled in terms of GBDP distance formula *d_5_*. The numbers above branches are GBDP pseudo-bootstrap support values > 60% from 100 replications, with an average branch support of 89.8%. The tree was rooted at the midpoint.

Table 1. Pairwise comparisons of *Pantoea sp.* strain MHSD4 vs. type strain genomes

| **Query strain** | **Subject strain** | **dDDH (d0, in %)** | **C.I. (d0, in %)** | **dDDH (d4, in %)** | **C.I. (d4, in %)** | **dDDH (d6, in %)** | **C.I. (d6, in %)** | **G+C content difference (in %)** |
| --- | --- | --- | --- | --- | --- | --- | --- | --- |
| *'Pantoea sp.* strain MHSD4' | *Pantoea hericii* JZB 2120024T | 81,3 | [77.4 - 84.7] | 93,9 | [92.1 - 95.3] | 86,3 | [83.2 - 88.9] | 0,44 |
| *'Pantoea sp. strain* MHSD4' | *Pantoea eucalypti LMG 24197* | 95 | [92.7 - 96.6] | 93,8 | [92.0 - 95.2] | 96,6 | [95.0 - 97.7] | 0,11 |
| *'Pantoea sp. strain MHSD4'* | *Pantoea agglomerans NBRC 102470* | 76,9 | [72.9 - 80.4] | 41,5 | [39.0 - 44.0] | 69,6 | [66.2 - 72.8] | 0,96 |
| *'Pantoea sp. strain MHSD4'* | *Curtobacterium plantarum LMG 16222* | 73,7 | [69.8 - 77.4] | 41,4 | [38.9 - 44.0] | 67,1 | [63.7 - 70.4] | 0,9 |
| *'Pantoea sp. strain MHSD4'* | *Pantoea pleuroti JZB 2120015* | 75,8 | [71.8 - 79.4] | 41,2 | [38.7 - 43.8] | 68,7 | [65.3 - 71.9] | 0,9 |
| *'Pantoea sp. strain MHSD4'* | *Pantoea vagans LMG 24199* | 73,6 | [69.7 - 77.3] | 40,5 | [38.0 - 43.0] | 66,6 | [63.2 - 69.9] | 1,18 |
| *'Pantoea sp. strain MHSD4'* | *Pantoea alfalfae CQ10T* | 73 | [69.0 - 76.6] | 40,2 | [37.7 - 42.7] | 66 | [62.6 - 69.2] | 0,82 |
| *'Pantoea sp. strain MHSD4'* | *Pantoea deleyi LMG 24200* | 62,8 | [59.1 - 66.4] | 33,1 | [30.6 - 35.6] | 54,9 | [51.7 - 58.0] | 3,51 |
| *'Pantoea sp. strain MHSD4'* | *Pantoea anthophila LMG 2558* | 68,1 | [64.2 - 71.8] | 32,9 | [30.5 - 35.4] | 58,6 | [55.4 - 61.7] | 2,6 |
| *'Pantoea sp. strain MHSD4'* | *Pantoea conspicua LMG 24534* | 59,5 | [55.9 - 63.1] | 28,5 | [26.1 - 31.0] | 50 | [47.0 - 53.1] | 1,39 |
| *'Pantoea sp. strain MHSD4'* | *Pantoea brenneri LMG 5343* | 57,1 | [53.6 - 60.7] | 28,4 | [26.0 - 30.9] | 48,4 | [45.4 - 51.4] | 1,58 |
| *'Pantoea sp. strain MHSD4'* | *Pantoea allii LMG 24248* | 30,2 | [26.8 - 33.8] | 22,4 | [20.2 - 24.9] | 27,3 | [24.4 - 30.4] | 1,18 |
| *'Pantoea sp. strain MHSD4'* | *Pantoea ananatis LMG 2665* | 31,2 | [27.8 - 34.8] | 22,3 | [20.1 - 24.8] | 28 | [25.1 - 31.1] | 0,76 |
| *'Pantoea sp. strain MHSD4'* | *Dryocola clanedunensis H11S18* | 15 | [12.1 - 18.4] | 19,9 | [17.7 - 22.3] | 15,1 | [12.6 - 18.0] | 0,31 |


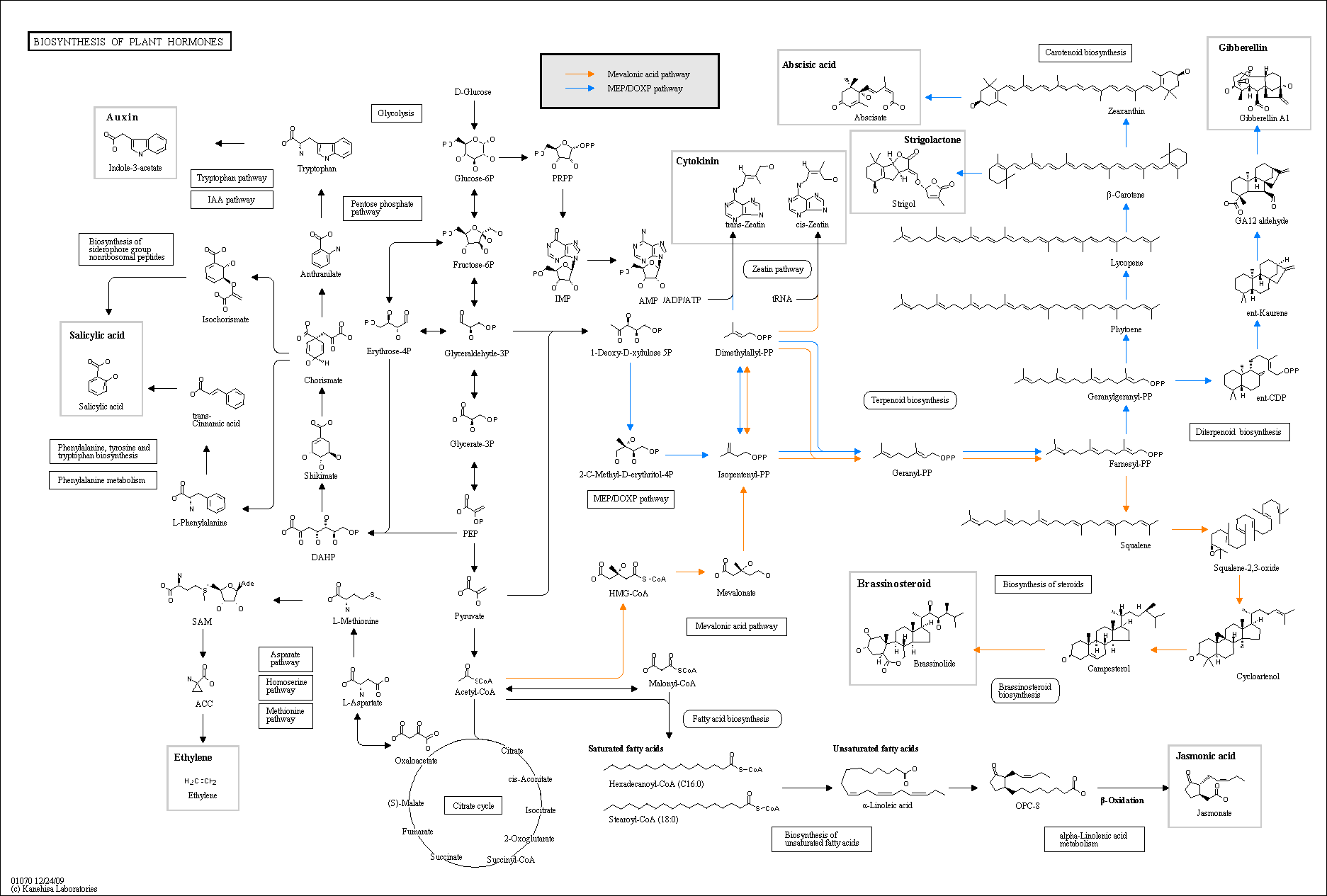


Figure 3. Biosynthesis pathways of plant hormones in Pantoea sp. strain MHSD4 aligned against Pantoea stewartii DC283. Arrows represent Mevalonic acid pathway and blue arrows represent MEP/DOXP pathway


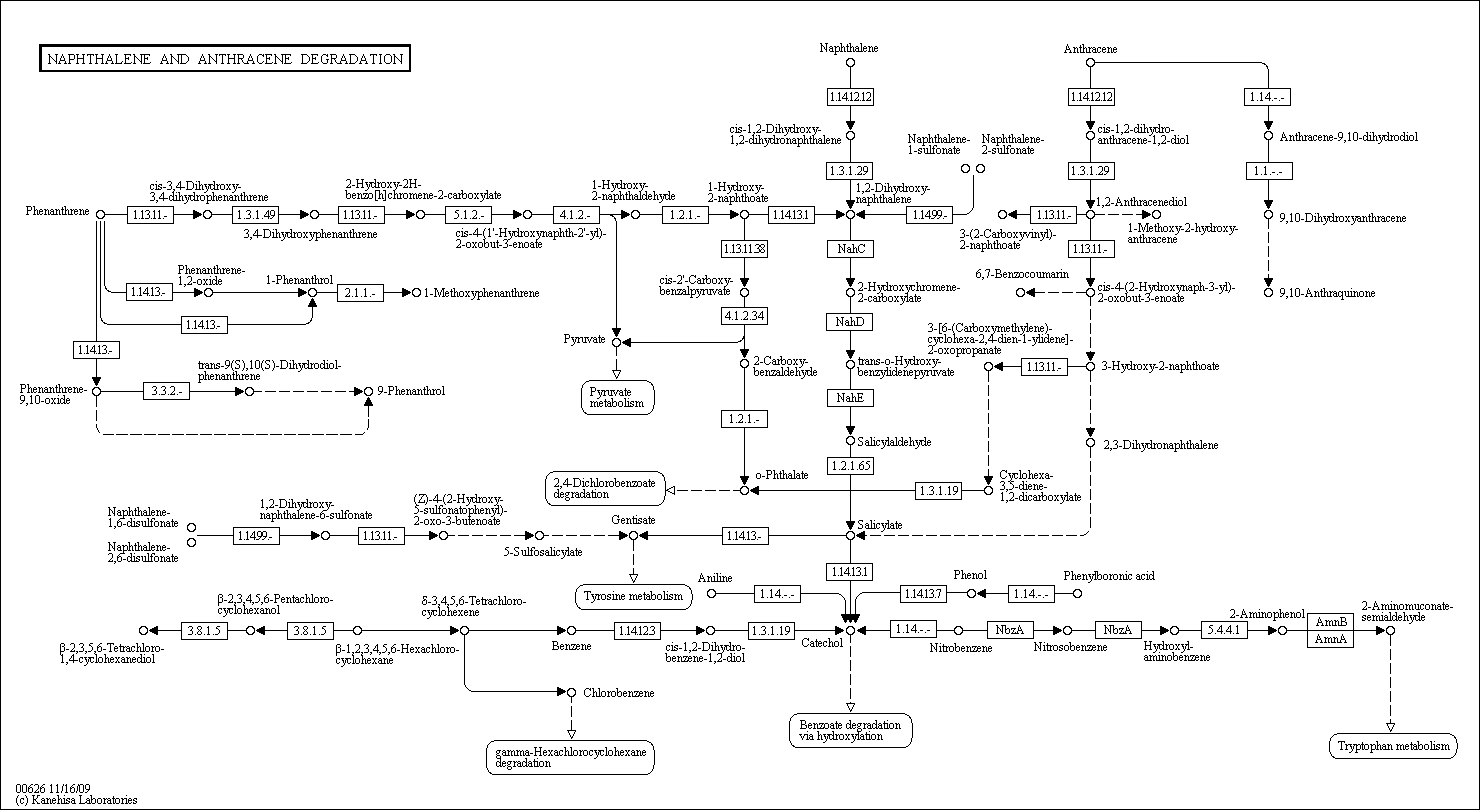


Figure 4. Biosynthesis pathways of naphthalene and anthracene degradation in *Pantoea sp.* strain MHSD4 aligned against *Pantoea stewartii* DC283.

Table 2. Genomic islands genes within *Pantoea* sp. strain MHSD4 aligned against reference genome within *Pantoea ananatis* LMG 20103, complete genome. The genomic islands genes prediction methods are represented by the following colours: IslandPath-DIMOB (blue) and integrated detection (empty blocks).

| Prediction Method | Gene Name | Accnum | Product |
| --- | --- | --- | --- |
|  | LQK91_21945 | [None](http://www.ncbi.nlm.nih.gov/protein/None) |  |
|  | LQK91_21845 | [None](http://www.ncbi.nlm.nih.gov/protein/None) |  |
|  | LQK91_21850 | [None](http://www.ncbi.nlm.nih.gov/protein/None) |  |
|  | LQK91_21855 | [None](http://www.ncbi.nlm.nih.gov/protein/None) |  |
|  | LQK91_21860 | [None](http://www.ncbi.nlm.nih.gov/protein/None) |  |
|  | LQK91_21865 | [None](http://www.ncbi.nlm.nih.gov/protein/None) |  |
|  | LQK91_21575 | [None](http://www.ncbi.nlm.nih.gov/protein/None) |  |
|  | LQK91_21580 | [None](http://www.ncbi.nlm.nih.gov/protein/None) |  |
|  | LQK91_21585 | [None](http://www.ncbi.nlm.nih.gov/protein/None) |  |
|  | LQK91_21590 | [None](http://www.ncbi.nlm.nih.gov/protein/None) |  |
|  | LQK91_21595 | [None](http://www.ncbi.nlm.nih.gov/protein/None) |  |
|  | LQK91_21600 | [None](http://www.ncbi.nlm.nih.gov/protein/None) |  |
|  | LQK91_21875 | [None](http://www.ncbi.nlm.nih.gov/protein/None) |  |
|  | LQK91_21880 | [None](http://www.ncbi.nlm.nih.gov/protein/None) |  |
|  | LQK91_21925 | [None](http://www.ncbi.nlm.nih.gov/protein/None) |  |
|  | LQK91_21840 | [None](http://www.ncbi.nlm.nih.gov/protein/None) |  |
|  | LQK91_21775 | [None](http://www.ncbi.nlm.nih.gov/protein/None) |  |
|  | LQK91_21780 | [None](http://www.ncbi.nlm.nih.gov/protein/None) |  |
|  | LQK91_21785 | [None](http://www.ncbi.nlm.nih.gov/protein/None) |  |
|  | LQK91_21790 | [None](http://www.ncbi.nlm.nih.gov/protein/None) |  |
|  | LQK91_21795 | [None](http://www.ncbi.nlm.nih.gov/protein/None) |  |
|  | LQK91_21800 | [None](http://www.ncbi.nlm.nih.gov/protein/None) |  |
|  | LQK91_21635 | [None](http://www.ncbi.nlm.nih.gov/protein/None) |  |
|  | LQK91_21640 | [None](http://www.ncbi.nlm.nih.gov/protein/None) |  |
|  | LQK91_21645 | [None](http://www.ncbi.nlm.nih.gov/protein/None) |  |
|  | LQK91_21650 | [None](http://www.ncbi.nlm.nih.gov/protein/None) |  |
|  | vgrG | [None](http://www.ncbi.nlm.nih.gov/protein/None) |  |
|  | LQK91_21885 | [None](http://www.ncbi.nlm.nih.gov/protein/None) |  |
|  | LQK91_21890 | [None](http://www.ncbi.nlm.nih.gov/protein/None) |  |
|  | LQK91_21895 | [None](http://www.ncbi.nlm.nih.gov/protein/None) |  |
|  | LQK91_21900 | [None](http://www.ncbi.nlm.nih.gov/protein/None) |  |
|  | LQK91_21930 | [None](http://www.ncbi.nlm.nih.gov/protein/None) |  |
|  | LQK91_21975 | [None](http://www.ncbi.nlm.nih.gov/protein/None) |  |
|  | LQK91_21255 | [None](http://www.ncbi.nlm.nih.gov/protein/None) |  |
|  | LQK91_21260 | [None](http://www.ncbi.nlm.nih.gov/protein/None) |  |
|  | LQK91_21265 | [None](http://www.ncbi.nlm.nih.gov/protein/None) |  |
|  | LQK91_21270 | [None](http://www.ncbi.nlm.nih.gov/protein/None) |  |
|  | LQK91_21275 | [None](http://www.ncbi.nlm.nih.gov/protein/None) |  |
|  | LQK91_21280 | [None](http://www.ncbi.nlm.nih.gov/protein/None) |  |
|  | LQK91_21285 | [None](http://www.ncbi.nlm.nih.gov/protein/None) |  |
|  | LQK91_21290 | [None](http://www.ncbi.nlm.nih.gov/protein/None) |  |
|  | LQK91_21295 | [None](http://www.ncbi.nlm.nih.gov/protein/None) |  |
|  | LQK91_21300 | [None](http://www.ncbi.nlm.nih.gov/protein/None) |  |
|  | LQK91_21305 | [None](http://www.ncbi.nlm.nih.gov/protein/None) |  |
|  | LQK91_21310 | [None](http://www.ncbi.nlm.nih.gov/protein/None) |  |
|  | umuD | [None](http://www.ncbi.nlm.nih.gov/protein/None) |  |
|  | umuC | [None](http://www.ncbi.nlm.nih.gov/protein/None) | translesion error-prone DNA polymerase V subunit UmuC |
|  | LQK91_21325 | [None](http://www.ncbi.nlm.nih.gov/protein/None) |  |
|  | LQK91_21330 | [None](http://www.ncbi.nlm.nih.gov/protein/None) |  |
|  | LQK91_21335 | [None](http://www.ncbi.nlm.nih.gov/protein/None) |  |
|  | LQK91_21340 | [None](http://www.ncbi.nlm.nih.gov/protein/None) |  |
|  | LQK91_21345 | [None](http://www.ncbi.nlm.nih.gov/protein/None) |  |
|  | LQK91_21350 | [None](http://www.ncbi.nlm.nih.gov/protein/None) |  |
|  | LQK91_21355 | [None](http://www.ncbi.nlm.nih.gov/protein/None) |  |
|  | LQK91_21360 | [None](http://www.ncbi.nlm.nih.gov/protein/None) |  |
|  | umuC | [None](http://www.ncbi.nlm.nih.gov/protein/None) |  |
|  | LQK91_21370 | [None](http://www.ncbi.nlm.nih.gov/protein/None) |  |
|  | LQK91_21375 | [None](http://www.ncbi.nlm.nih.gov/protein/None) |  |
|  | LQK91_21380 | [None](http://www.ncbi.nlm.nih.gov/protein/None) |  |
|  | LQK91_21385 | [None](http://www.ncbi.nlm.nih.gov/protein/None) |  |
|  | LQK91_21390 | [None](http://www.ncbi.nlm.nih.gov/protein/None) |  |
|  | LQK91_21395 | [None](http://www.ncbi.nlm.nih.gov/protein/None) |  |
|  | LQK91_21400 | [None](http://www.ncbi.nlm.nih.gov/protein/None) |  |
|  | LQK91_21405 | [None](http://www.ncbi.nlm.nih.gov/protein/None) |  |
|  | LQK91_21410 | [None](http://www.ncbi.nlm.nih.gov/protein/None) |  |
|  | LQK91_21910 | [None](http://www.ncbi.nlm.nih.gov/protein/None) |  |
|  | LQK91_21915 | [None](http://www.ncbi.nlm.nih.gov/protein/None) |  |
|  | LQK91_21525 | [None](http://www.ncbi.nlm.nih.gov/protein/None) |  |
|  | LQK91_21530 | [None](http://www.ncbi.nlm.nih.gov/protein/None) |  |
|  | LQK91_21535 | [None](http://www.ncbi.nlm.nih.gov/protein/None) |  |
|  | LQK91_21540 | [None](http://www.ncbi.nlm.nih.gov/protein/None) |  |
|  | LQK91_21545 | [None](http://www.ncbi.nlm.nih.gov/protein/None) |  |
|  | LQK91_21550 | [None](http://www.ncbi.nlm.nih.gov/protein/None) |  |
|  | LQK91_21555 | [None](http://www.ncbi.nlm.nih.gov/protein/None) |  |
|  | LQK91_21560 | [None](http://www.ncbi.nlm.nih.gov/protein/None) |  |
|  | LQK91_21565 | [None](http://www.ncbi.nlm.nih.gov/protein/None) |  |
|  | LQK91_21570 | [None](http://www.ncbi.nlm.nih.gov/protein/None) |  |
|  | LQK91_21935 | [None](http://www.ncbi.nlm.nih.gov/protein/None) |  |
|  | LQK91_21940 | [None](http://www.ncbi.nlm.nih.gov/protein/None) |  |
|  | tssC | [None](http://www.ncbi.nlm.nih.gov/protein/None) |  |
|  | LQK91_07820 | [None](http://www.ncbi.nlm.nih.gov/protein/None) |  |
|  | LQK91_07825 | [None](http://www.ncbi.nlm.nih.gov/protein/None) |  |
|  | tssF | [None](http://www.ncbi.nlm.nih.gov/protein/None) |  |
|  | tssG | [None](http://www.ncbi.nlm.nih.gov/protein/None) |  |
|  | vgrG | [None](http://www.ncbi.nlm.nih.gov/protein/None) |  |
|  | LQK91_07845 | [None](http://www.ncbi.nlm.nih.gov/protein/None) |  |
|  | LQK91_07850 | [None](http://www.ncbi.nlm.nih.gov/protein/None) |  |
|  | tssA | [None](http://www.ncbi.nlm.nih.gov/protein/None) |  |
|  | LQK91_07860 | [None](http://www.ncbi.nlm.nih.gov/protein/None) |  |
|  | tssK | [None](http://www.ncbi.nlm.nih.gov/protein/None) |  |
|  | LQK91_07870 | [None](http://www.ncbi.nlm.nih.gov/protein/None) |  |
|  | LQK91_07875 | [None](http://www.ncbi.nlm.nih.gov/protein/None) |  |
|  | tssH | [None](http://www.ncbi.nlm.nih.gov/protein/None) |  |
|  | LQK91_07885 | [None](http://www.ncbi.nlm.nih.gov/protein/None) |  |
|  | LQK91_07890 | [None](http://www.ncbi.nlm.nih.gov/protein/None) |  |
|  | LQK91_07895 | [None](http://www.ncbi.nlm.nih.gov/protein/None) |  |
|  | LQK91_07900 | [None](http://www.ncbi.nlm.nih.gov/protein/None) |  |
|  | ddpX | [None](http://www.ncbi.nlm.nih.gov/protein/None) |  |
|  | LQK91_07910 | [None](http://www.ncbi.nlm.nih.gov/protein/None) |  |
|  | LQK91_07915 | [None](http://www.ncbi.nlm.nih.gov/protein/None) |  |
|  | LQK91_07920 | [None](http://www.ncbi.nlm.nih.gov/protein/None) |  |
|  | corA | [None](http://www.ncbi.nlm.nih.gov/protein/None) |  |
|  | LQK91_07930 | [None](http://www.ncbi.nlm.nih.gov/protein/None) |  |
|  | LQK91_07935 | [None](http://www.ncbi.nlm.nih.gov/protein/None) |  |
|  | LQK91_07940 | [None](http://www.ncbi.nlm.nih.gov/protein/None) |  |
|  | LQK91_07945 | [None](http://www.ncbi.nlm.nih.gov/protein/None) |  |
|  | LQK91_07950 | [None](http://www.ncbi.nlm.nih.gov/protein/None) |  |
|  | LQK91_07955 | [None](http://www.ncbi.nlm.nih.gov/protein/None) |  |
|  | LQK91_07960 | [None](http://www.ncbi.nlm.nih.gov/protein/None) |  |
|  | fliN | [None](http://www.ncbi.nlm.nih.gov/protein/None) |  |
|  | fliO | [None](http://www.ncbi.nlm.nih.gov/protein/None) |  |
|  | fliP | [None](http://www.ncbi.nlm.nih.gov/protein/None) |  |
|  | fliQ | [None](http://www.ncbi.nlm.nih.gov/protein/None) |  |
|  | fliR | [None](http://www.ncbi.nlm.nih.gov/protein/None) |  |
|  | rcsA | [None](http://www.ncbi.nlm.nih.gov/protein/None) |  |
|  | LQK91_03160 | [None](http://www.ncbi.nlm.nih.gov/protein/None) |  |
|  | dsrB | [None](http://www.ncbi.nlm.nih.gov/protein/None) |  |
|  | LQK91_03150 | [None](http://www.ncbi.nlm.nih.gov/protein/None) |  |
|  | LQK91_03145 | [None](http://www.ncbi.nlm.nih.gov/protein/None) |  |
|  | LQK91_03140 | [None](http://www.ncbi.nlm.nih.gov/protein/None) |  |
|  | LQK91_03135 | [None](http://www.ncbi.nlm.nih.gov/protein/None) |  |
|  | LQK91_03130 | [None](http://www.ncbi.nlm.nih.gov/protein/None) |  |
|  | LQK91_03125 | [None](http://www.ncbi.nlm.nih.gov/protein/None) |  |
|  | LQK91_03120 | [None](http://www.ncbi.nlm.nih.gov/protein/None) |  |
|  | vsr | [None](http://www.ncbi.nlm.nih.gov/protein/None) |  |
|  | dcm | [None](http://www.ncbi.nlm.nih.gov/protein/None) |  |
|  | LQK91_03105 | [None](http://www.ncbi.nlm.nih.gov/protein/None) |  |
|  | LQK91_03100 | [None](http://www.ncbi.nlm.nih.gov/protein/None) |  |
|  | LQK91_03095 | [None](http://www.ncbi.nlm.nih.gov/protein/None) |  |
|  | mtfA | [None](http://www.ncbi.nlm.nih.gov/protein/None) |  |
|  | LQK91_03080 | [None](http://www.ncbi.nlm.nih.gov/protein/None) |  |
|  | LQK91_03075 | [None](http://www.ncbi.nlm.nih.gov/protein/None) |  |
|  | LQK91_03070 | [None](http://www.ncbi.nlm.nih.gov/protein/None) |  |
|  | LQK91_03065 | [None](http://www.ncbi.nlm.nih.gov/protein/None) |  |
|  | LQK91_03060 | [None](http://www.ncbi.nlm.nih.gov/protein/None) |  |
|  | LQK91_03055 | [None](http://www.ncbi.nlm.nih.gov/protein/None) |  |
|  | LQK91_03050 | [None](http://www.ncbi.nlm.nih.gov/protein/None) |  |
|  | LQK91_03045 | [None](http://www.ncbi.nlm.nih.gov/protein/None) |  |
|  | LQK91_03040 | [None](http://www.ncbi.nlm.nih.gov/protein/None) |  |
|  | LQK91_03035 | [None](http://www.ncbi.nlm.nih.gov/protein/None) |  |
|  | LQK91_03030 | [None](http://www.ncbi.nlm.nih.gov/protein/None) |  |
|  | LQK91_03025 | [None](http://www.ncbi.nlm.nih.gov/protein/None) |  |
|  | LQK91_03020 | [None](http://www.ncbi.nlm.nih.gov/protein/None) |  |
|  | LQK91_03015 | [None](http://www.ncbi.nlm.nih.gov/protein/None) |  |
|  | LQK91_03010 | [None](http://www.ncbi.nlm.nih.gov/protein/None) |  |
|  | LQK91_03005 | [None](http://www.ncbi.nlm.nih.gov/protein/None) |  |
|  | LQK91_03000 | [None](http://www.ncbi.nlm.nih.gov/protein/None) |  |
|  | LQK91_02995 | [None](http://www.ncbi.nlm.nih.gov/protein/None) |  |
|  | LQK91_02990 | [None](http://www.ncbi.nlm.nih.gov/protein/None) |  |
|  | LQK91_02985 | [None](http://www.ncbi.nlm.nih.gov/protein/None) |  |
|  | LQK91_02980 | [None](http://www.ncbi.nlm.nih.gov/protein/None) |  |
|  | LQK91_02975 | [None](http://www.ncbi.nlm.nih.gov/protein/None) | DNA-binding protein |
|  | LQK91_02970 | [None](http://www.ncbi.nlm.nih.gov/protein/None) |  |
|  | LQK91_02965 | [None](http://www.ncbi.nlm.nih.gov/protein/None) |  |
|  | LQK91_02960 | [None](http://www.ncbi.nlm.nih.gov/protein/None) |  |
|  | LQK91_02955 | [None](http://www.ncbi.nlm.nih.gov/protein/None) |  |
|  | LQK91_02950 | [None](http://www.ncbi.nlm.nih.gov/protein/None) |  |
|  | LQK91_02945 | [None](http://www.ncbi.nlm.nih.gov/protein/None) |  |
|  | LQK91_02940 | [None](http://www.ncbi.nlm.nih.gov/protein/None) |  |
|  | LQK91_02935 | [None](http://www.ncbi.nlm.nih.gov/protein/None) |  |
|  | LQK91_02930 | [None](http://www.ncbi.nlm.nih.gov/protein/None) |  |
|  | LQK91_02925 | [None](http://www.ncbi.nlm.nih.gov/protein/None) |  |
|  | LQK91_02920 | [None](http://www.ncbi.nlm.nih.gov/protein/None) |  |
|  | LQK91_02915 | [None](http://www.ncbi.nlm.nih.gov/protein/None) |  |
|  | LQK91_02910 | [None](http://www.ncbi.nlm.nih.gov/protein/None) |  |
|  | LQK91_02905 | [None](http://www.ncbi.nlm.nih.gov/protein/None) |  |
|  | LQK91_02900 | [None](http://www.ncbi.nlm.nih.gov/protein/None) |  |
|  | LQK91_02895 | [None](http://www.ncbi.nlm.nih.gov/protein/None) |  |
|  | LQK91_02890 | [None](http://www.ncbi.nlm.nih.gov/protein/None) |  |
|  | LQK91_02885 | [None](http://www.ncbi.nlm.nih.gov/protein/None) |  |
|  | LQK91_02880 | [None](http://www.ncbi.nlm.nih.gov/protein/None) |  |
|  | LQK91_00475 | [None](http://www.ncbi.nlm.nih.gov/protein/None) |  |
|  | LQK91_00470 | [None](http://www.ncbi.nlm.nih.gov/protein/None) |  |
|  | acpS | [None](http://www.ncbi.nlm.nih.gov/protein/None) |  |
|  | pdxJ | [None](http://www.ncbi.nlm.nih.gov/protein/None) |  |
|  | recO | [None](http://www.ncbi.nlm.nih.gov/protein/None) |  |
|  | era | [None](http://www.ncbi.nlm.nih.gov/protein/None) |  |
|  | rnc | [None](http://www.ncbi.nlm.nih.gov/protein/None) |  |
|  | lepB | [None](http://www.ncbi.nlm.nih.gov/protein/None) |  |
|  | lepA | [None](http://www.ncbi.nlm.nih.gov/protein/None) |  |
|  | rseC | [None](http://www.ncbi.nlm.nih.gov/protein/None) |  |
|  | rseB | [None](http://www.ncbi.nlm.nih.gov/protein/None) |  |
|  | rseA | [None](http://www.ncbi.nlm.nih.gov/protein/None) |  |
|  | rpoE | [None](http://www.ncbi.nlm.nih.gov/protein/None) |  |
|  | nadB | [None](http://www.ncbi.nlm.nih.gov/protein/None) |  |
|  | LQK91_00405 | [None](http://www.ncbi.nlm.nih.gov/protein/None) |  |
|  | srmB | [None](http://www.ncbi.nlm.nih.gov/protein/None) |  |
|  | grcA | [None](http://www.ncbi.nlm.nih.gov/protein/None) |  |
|  | ung | [None](http://www.ncbi.nlm.nih.gov/protein/None) |  |
|  | grpE | [None](http://www.ncbi.nlm.nih.gov/protein/None) |  |
|  | nadK | [None](http://www.ncbi.nlm.nih.gov/protein/None) |  |
|  | recN | [None](http://www.ncbi.nlm.nih.gov/protein/None) |  |
|  | bamE | [None](http://www.ncbi.nlm.nih.gov/protein/None) |  |
|  | LQK91_00365 | [None](http://www.ncbi.nlm.nih.gov/protein/None) |  |
|  | LQK91_00360 | [None](http://www.ncbi.nlm.nih.gov/protein/None) |  |
|  | smpB | [None](http://www.ncbi.nlm.nih.gov/protein/None) |  |
|  | LQK91_00345 | [None](http://www.ncbi.nlm.nih.gov/protein/None) |  |
|  | LQK91_00340 | [None](http://www.ncbi.nlm.nih.gov/protein/None) |  |
|  | LQK91_00335 | [None](http://www.ncbi.nlm.nih.gov/protein/None) |  |
|  | LQK91_00330 | [None](http://www.ncbi.nlm.nih.gov/protein/None) |  |
|  | LQK91_00325 | [None](http://www.ncbi.nlm.nih.gov/protein/None) |  |
|  | LQK91_00320 | [None](http://www.ncbi.nlm.nih.gov/protein/None) |  |
|  | LQK91_00315 | [None](http://www.ncbi.nlm.nih.gov/protein/None) |  |
|  | LQK91_00310 | [None](http://www.ncbi.nlm.nih.gov/protein/None) |  |
|  | LQK91_00305 | [None](http://www.ncbi.nlm.nih.gov/protein/None) |  |
|  | LQK91_00300 | [None](http://www.ncbi.nlm.nih.gov/protein/None) |  |
|  | LQK91_00295 | [None](http://www.ncbi.nlm.nih.gov/protein/None) |  |
|  | LQK91_00290 | [None](http://www.ncbi.nlm.nih.gov/protein/None) |  |
|  | LQK91_00285 | [None](http://www.ncbi.nlm.nih.gov/protein/None) |  |
|  | LQK91_00280 | [None](http://www.ncbi.nlm.nih.gov/protein/None) |  |
|  | LQK91_00275 | [None](http://www.ncbi.nlm.nih.gov/protein/None) | integrase arm-type DNA-binding domain-containing protein |
|  | LQK91_00270 | [None](http://www.ncbi.nlm.nih.gov/protein/None) |  |
|  | LQK91_00265 | [None](http://www.ncbi.nlm.nih.gov/protein/None) |  |
|  | LQK91_00260 | [None](http://www.ncbi.nlm.nih.gov/protein/None) |  |
|  | LQK91_00255 | [None](http://www.ncbi.nlm.nih.gov/protein/None) |  |
|  | LQK91_00250 | [None](http://www.ncbi.nlm.nih.gov/protein/None) |  |
|  | LQK91_00245 | [None](http://www.ncbi.nlm.nih.gov/protein/None) |  |
|  | LQK91_00240 | [None](http://www.ncbi.nlm.nih.gov/protein/None) |  |
|  | LQK91_00235 | [None](http://www.ncbi.nlm.nih.gov/protein/None) |  |
|  | LQK91_00230 | [None](http://www.ncbi.nlm.nih.gov/protein/None) |  |
|  | oxlT | [None](http://www.ncbi.nlm.nih.gov/protein/None) |  |
|  | cirA | [None](http://www.ncbi.nlm.nih.gov/protein/None) |  |
|  | LQK91_00215 | [None](http://www.ncbi.nlm.nih.gov/protein/None) |  |
|  | LQK91_00210 | [None](http://www.ncbi.nlm.nih.gov/protein/None) |  |
|  | LQK91_00205 | [None](http://www.ncbi.nlm.nih.gov/protein/None) |  |
|  | LQK91_00200 | [None](http://www.ncbi.nlm.nih.gov/protein/None) |  |
|  | LQK91_00195 | [None](http://www.ncbi.nlm.nih.gov/protein/None) |  |
|  | LQK91_00190 | [None](http://www.ncbi.nlm.nih.gov/protein/None) |  |
|  | LQK91_00185 | [None](http://www.ncbi.nlm.nih.gov/protein/None) |  |
|  | alsK | [None](http://www.ncbi.nlm.nih.gov/protein/None) |  |
|  | LQK91_00175 | [None](http://www.ncbi.nlm.nih.gov/protein/None) |  |
|  | LQK91_00170 | [None](http://www.ncbi.nlm.nih.gov/protein/None) |  |
|  | LQK91_00165 | [None](http://www.ncbi.nlm.nih.gov/protein/None) |  |
|  | LQK91_00160 | [None](http://www.ncbi.nlm.nih.gov/protein/None) |  |
|  | mgtA | [None](http://www.ncbi.nlm.nih.gov/protein/None) |  |
|  | alaS | [None](http://www.ncbi.nlm.nih.gov/protein/None) |  |
|  | LQK91_16900 | [None](http://www.ncbi.nlm.nih.gov/protein/None) |  |
|  | recA | [None](http://www.ncbi.nlm.nih.gov/protein/None) |  |
|  | pncC | [None](http://www.ncbi.nlm.nih.gov/protein/None) |  |
|  | tam | [None](http://www.ncbi.nlm.nih.gov/protein/None) |  |
|  | LQK91_16880 | [None](http://www.ncbi.nlm.nih.gov/protein/None) |  |
|  | LQK91_16875 | [None](http://www.ncbi.nlm.nih.gov/protein/None) |  |
|  | mltB | [None](http://www.ncbi.nlm.nih.gov/protein/None) |  |
|  | narI | [None](http://www.ncbi.nlm.nih.gov/protein/None) |  |
|  | narJ | [None](http://www.ncbi.nlm.nih.gov/protein/None) |  |
|  | narH | [None](http://www.ncbi.nlm.nih.gov/protein/None) |  |
|  | LQK91_16850 | [None](http://www.ncbi.nlm.nih.gov/protein/None) |  |
|  | LQK91_16845 | [None](http://www.ncbi.nlm.nih.gov/protein/None) |  |
|  | narL | [None](http://www.ncbi.nlm.nih.gov/protein/None) |  |
|  | LQK91_16835 | [None](http://www.ncbi.nlm.nih.gov/protein/None) |  |
|  | LQK91_16830 | [None](http://www.ncbi.nlm.nih.gov/protein/None) |  |
|  | LQK91_16825 | [None](http://www.ncbi.nlm.nih.gov/protein/None) |  |
|  | LQK91_16820 | [None](http://www.ncbi.nlm.nih.gov/protein/None) |  |
|  | LQK91_16815 | [None](http://www.ncbi.nlm.nih.gov/protein/None) |  |
|  | LQK91_16810 | [None](http://www.ncbi.nlm.nih.gov/protein/None) |  |
|  | LQK91_16805 | [None](http://www.ncbi.nlm.nih.gov/protein/None) |  |
|  | LQK91_16800 | [None](http://www.ncbi.nlm.nih.gov/protein/None) |  |
|  | LQK91_16795 | [None](http://www.ncbi.nlm.nih.gov/protein/None) |  |
|  | radC | [None](http://www.ncbi.nlm.nih.gov/protein/None) |  |
|  | LQK91_16785 | [None](http://www.ncbi.nlm.nih.gov/protein/None) |  |
|  | LQK91_16780 | [None](http://www.ncbi.nlm.nih.gov/protein/None) |  |
|  | LQK91_16775 | [None](http://www.ncbi.nlm.nih.gov/protein/None) |  |
|  | LQK91_16770 | [None](http://www.ncbi.nlm.nih.gov/protein/None) |  |
|  | LQK91_16765 | [None](http://www.ncbi.nlm.nih.gov/protein/None) |  |
|  | LQK91_16760 | [None](http://www.ncbi.nlm.nih.gov/protein/None) |  |
|  | LQK91_16755 | [None](http://www.ncbi.nlm.nih.gov/protein/None) |  |
|  | LQK91_16750 | [None](http://www.ncbi.nlm.nih.gov/protein/None) |  |
|  | LQK91_16745 | [None](http://www.ncbi.nlm.nih.gov/protein/None) |  |
|  | LQK91_16740 | [None](http://www.ncbi.nlm.nih.gov/protein/None) |  |
|  | LQK91_16735 | [None](http://www.ncbi.nlm.nih.gov/protein/None) |  |
|  | LQK91_16730 | [None](http://www.ncbi.nlm.nih.gov/protein/None) |  |
|  | mutS | [None](http://www.ncbi.nlm.nih.gov/protein/None) |  |
|  | rpoS | [None](http://www.ncbi.nlm.nih.gov/protein/None) |  |
|  | nlpD | [None](http://www.ncbi.nlm.nih.gov/protein/None) |  |
|  | LQK91_16710 | [None](http://www.ncbi.nlm.nih.gov/protein/None) |  |
|  | surE | [None](http://www.ncbi.nlm.nih.gov/protein/None) |  |
|  | truD | [None](http://www.ncbi.nlm.nih.gov/protein/None) |  |
|  | ispF | [None](http://www.ncbi.nlm.nih.gov/protein/None) |  |
|  | ispD | [None](http://www.ncbi.nlm.nih.gov/protein/None) |  |
|  | ftsB | [None](http://www.ncbi.nlm.nih.gov/protein/None) |  |
|  | LQK91_16680 | [None](http://www.ncbi.nlm.nih.gov/protein/None) |  |
|  | cysC | [None](http://www.ncbi.nlm.nih.gov/protein/None) |  |
|  | cysN | [None](http://www.ncbi.nlm.nih.gov/protein/None) |  |
|  | cysD | [None](http://www.ncbi.nlm.nih.gov/protein/None) |  |
|  | cysG | [None](http://www.ncbi.nlm.nih.gov/protein/None) |  |
|  | LQK91_16655 | [None](http://www.ncbi.nlm.nih.gov/protein/None) |  |
|  | LQK91_16650 | [None](http://www.ncbi.nlm.nih.gov/protein/None) |  |
|  | LQK91_16645 | [None](http://www.ncbi.nlm.nih.gov/protein/None) |  |
|  | cysI | [None](http://www.ncbi.nlm.nih.gov/protein/None) |  |
|  | LQK91_15540 | [None](http://www.ncbi.nlm.nih.gov/protein/None) |  |
|  | LQK91_15535 | [None](http://www.ncbi.nlm.nih.gov/protein/None) |  |
|  | LQK91_15530 | [None](http://www.ncbi.nlm.nih.gov/protein/None) |  |
|  | bacA | [None](http://www.ncbi.nlm.nih.gov/protein/None) |  |
|  | folB | [None](http://www.ncbi.nlm.nih.gov/protein/None) |  |
|  | plsY | [None](http://www.ncbi.nlm.nih.gov/protein/None) |  |
|  | tsaD | [None](http://www.ncbi.nlm.nih.gov/protein/None) |  |
|  | rpsU | [None](http://www.ncbi.nlm.nih.gov/protein/None) |  |
|  | dnaG | [None](http://www.ncbi.nlm.nih.gov/protein/None) |  |
|  | rpoD | [None](http://www.ncbi.nlm.nih.gov/protein/None) |  |
|  | mug | [None](http://www.ncbi.nlm.nih.gov/protein/None) |  |
|  | LQK91_15485 | [None](http://www.ncbi.nlm.nih.gov/protein/None) |  |
|  | LQK91_15480 | [None](http://www.ncbi.nlm.nih.gov/protein/None) |  |
|  | LQK91_15470 | [None](http://www.ncbi.nlm.nih.gov/protein/None) |  |
|  | LQK91_15465 | [None](http://www.ncbi.nlm.nih.gov/protein/None) |  |
|  | LQK91_15460 | [None](http://www.ncbi.nlm.nih.gov/protein/None) |  |
|  | LQK91_15455 | [None](http://www.ncbi.nlm.nih.gov/protein/None) |  |
|  | LQK91_20155 | [None](http://www.ncbi.nlm.nih.gov/protein/None) |  |
|  | LQK91_20150 | [None](http://www.ncbi.nlm.nih.gov/protein/None) |  |
|  | LQK91_20145 | [None](http://www.ncbi.nlm.nih.gov/protein/None) | outer membrane protein transport protein |
|  | LQK91_20140 | [None](http://www.ncbi.nlm.nih.gov/protein/None) |  |
|  | LQK91_20135 | [None](http://www.ncbi.nlm.nih.gov/protein/None) |  |
|  | LQK91_20130 | [None](http://www.ncbi.nlm.nih.gov/protein/None) |  |
|  | LQK91_20125 | [None](http://www.ncbi.nlm.nih.gov/protein/None) |  |
|  | LQK91_20120 | [None](http://www.ncbi.nlm.nih.gov/protein/None) |  |
|  | LQK91_20115 | [None](http://www.ncbi.nlm.nih.gov/protein/None) |  |
|  | LQK91_20110 | [None](http://www.ncbi.nlm.nih.gov/protein/None) |  |
|  | LQK91_20105 | [None](http://www.ncbi.nlm.nih.gov/protein/None) |  |
|  | LQK91_20100 | [None](http://www.ncbi.nlm.nih.gov/protein/None) |  |
|  | LQK91_20095 | [None](http://www.ncbi.nlm.nih.gov/protein/None) |  |
|  | LQK91_20090 | [None](http://www.ncbi.nlm.nih.gov/protein/None) |  |
|  | LQK91_20085 | [None](http://www.ncbi.nlm.nih.gov/protein/None) |  |
|  | LQK91_20080 | [None](http://www.ncbi.nlm.nih.gov/protein/None) |  |
|  | LQK91_20075 | [None](http://www.ncbi.nlm.nih.gov/protein/None) |  |
|  | LQK91_20070 | [None](http://www.ncbi.nlm.nih.gov/protein/None) |  |
|  | LQK91_20065 | [None](http://www.ncbi.nlm.nih.gov/protein/None) |  |
|  | LQK91_20060 | [None](http://www.ncbi.nlm.nih.gov/protein/None) |  |
|  | LQK91_20055 | [None](http://www.ncbi.nlm.nih.gov/protein/None) |  |
|  | ddlA | [None](http://www.ncbi.nlm.nih.gov/protein/None) |  |
|  | LQK91_20045 | [None](http://www.ncbi.nlm.nih.gov/protein/None) |  |
|  | LQK91_20040 | [None](http://www.ncbi.nlm.nih.gov/protein/None) |  |
|  | LQK91_20035 | [None](http://www.ncbi.nlm.nih.gov/protein/None) |  |
|  | LQK91_20030 | [None](http://www.ncbi.nlm.nih.gov/protein/None) |  |
|  | LQK91_20025 | [None](http://www.ncbi.nlm.nih.gov/protein/None) |  |
|  | LQK91_20020 | [None](http://www.ncbi.nlm.nih.gov/protein/None) |  |
|  | rlmG | [None](http://www.ncbi.nlm.nih.gov/protein/None) |  |
|  | slmA | [None](http://www.ncbi.nlm.nih.gov/protein/None) |  |
|  | pyrE | [None](http://www.ncbi.nlm.nih.gov/protein/None) |  |
|  | rph | [None](http://www.ncbi.nlm.nih.gov/protein/None) |  |
|  | LQK91_21050 | [None](http://www.ncbi.nlm.nih.gov/protein/None) |  |
|  | LQK91_21055 | [None](http://www.ncbi.nlm.nih.gov/protein/None) |  |
|  | LQK91_21060 | [None](http://www.ncbi.nlm.nih.gov/protein/None) |  |
|  | LQK91_21065 | [None](http://www.ncbi.nlm.nih.gov/protein/None) |  |
|  | LQK91_21070 | [None](http://www.ncbi.nlm.nih.gov/protein/None) |  |
|  | LQK91_21075 | [None](http://www.ncbi.nlm.nih.gov/protein/None) |  |
|  | LQK91_21080 | [None](http://www.ncbi.nlm.nih.gov/protein/None) |  |
|  | LQK91_21085 | [None](http://www.ncbi.nlm.nih.gov/protein/None) |  |
|  | LQK91_21090 | [None](http://www.ncbi.nlm.nih.gov/protein/None) |  |
|  | LQK91_21095 | [None](http://www.ncbi.nlm.nih.gov/protein/None) |  |
|  | lpxP | [None](http://www.ncbi.nlm.nih.gov/protein/None) |  |
|  | LQK91_21105 | [None](http://www.ncbi.nlm.nih.gov/protein/None) |  |
|  | LQK91_21110 | [None](http://www.ncbi.nlm.nih.gov/protein/None) |  |
|  | LQK91_21115 | [None](http://www.ncbi.nlm.nih.gov/protein/None) |  |
|  | LQK91_14675 | [None](http://www.ncbi.nlm.nih.gov/protein/None) |  |
|  | LQK91_14680 | [None](http://www.ncbi.nlm.nih.gov/protein/None) |  |
|  | LQK91_14685 | [None](http://www.ncbi.nlm.nih.gov/protein/None) |  |
|  | LQK91_14690 | [None](http://www.ncbi.nlm.nih.gov/protein/None) |  |
|  | LQK91_14695 | [None](http://www.ncbi.nlm.nih.gov/protein/None) |  |
|  | LQK91_14700 | [None](http://www.ncbi.nlm.nih.gov/protein/None) |  |
|  | LQK91_14705 | [None](http://www.ncbi.nlm.nih.gov/protein/None) |  |
|  | gstA | [None](http://www.ncbi.nlm.nih.gov/protein/None) |  |
|  | LQK91_14715 | [None](http://www.ncbi.nlm.nih.gov/protein/None) |  |
|  | LQK91_14720 | [None](http://www.ncbi.nlm.nih.gov/protein/None) |  |
|  | LQK91_14725 | [None](http://www.ncbi.nlm.nih.gov/protein/None) |  |
|  | LQK91_14730 | [None](http://www.ncbi.nlm.nih.gov/protein/None) |  |
|  | LQK91_14735 | [None](http://www.ncbi.nlm.nih.gov/protein/None) |  |
|  | LQK91_14740 | [None](http://www.ncbi.nlm.nih.gov/protein/None) |  |
|  | LQK91_14745 | [None](http://www.ncbi.nlm.nih.gov/protein/None) |  |
|  | LQK91_14750 | [None](http://www.ncbi.nlm.nih.gov/protein/None) |  |
|  | LQK91_14755 | [None](http://www.ncbi.nlm.nih.gov/protein/None) |  |
|  | LQK91_14760 | [None](http://www.ncbi.nlm.nih.gov/protein/None) |  |
|  | LQK91_14765 | [None](http://www.ncbi.nlm.nih.gov/protein/None) |  |
|  | LQK91_14770 | [None](http://www.ncbi.nlm.nih.gov/protein/None) |  |
|  | LQK91_14775 | [None](http://www.ncbi.nlm.nih.gov/protein/None) |  |
|  | LQK91_14780 | [None](http://www.ncbi.nlm.nih.gov/protein/None) |  |
|  | LQK91_14785 | [None](http://www.ncbi.nlm.nih.gov/protein/None) |  |
|  | LQK91_14790 | [None](http://www.ncbi.nlm.nih.gov/protein/None) |  |
|  | LQK91_14795 | [None](http://www.ncbi.nlm.nih.gov/protein/None) |  |
|  | LQK91_14800 | [None](http://www.ncbi.nlm.nih.gov/protein/None) |  |
|  | LQK91_14805 | [None](http://www.ncbi.nlm.nih.gov/protein/None) |  |
|  | LQK91_14810 | [None](http://www.ncbi.nlm.nih.gov/protein/None) |  |
|  | LQK91_14815 | [None](http://www.ncbi.nlm.nih.gov/protein/None) |  |
|  | LQK91_14820 | [None](http://www.ncbi.nlm.nih.gov/protein/None) |  |
|  | LQK91_14825 | [None](http://www.ncbi.nlm.nih.gov/protein/None) |  |
|  | LQK91_14830 | [None](http://www.ncbi.nlm.nih.gov/protein/None) |  |
|  | LQK91_14835 | [None](http://www.ncbi.nlm.nih.gov/protein/None) |  |
|  | LQK91_14840 | [None](http://www.ncbi.nlm.nih.gov/protein/None) |  |
|  | LQK91_14845 | [None](http://www.ncbi.nlm.nih.gov/protein/None) |  |
|  | LQK91_14850 | [None](http://www.ncbi.nlm.nih.gov/protein/None) |  |
|  | LQK91_14855 | [None](http://www.ncbi.nlm.nih.gov/protein/None) |  |
|  | LQK91_14860 | [None](http://www.ncbi.nlm.nih.gov/protein/None) |  |
|  | LQK91_14865 | [None](http://www.ncbi.nlm.nih.gov/protein/None) |  |
|  | hemH | [None](http://www.ncbi.nlm.nih.gov/protein/None) |  |
|  | LQK91_14875 | [None](http://www.ncbi.nlm.nih.gov/protein/None) | DUF2778 domain-containing protein |
|  | LQK91_14880 | [None](http://www.ncbi.nlm.nih.gov/protein/None) |  |
|  | LQK91_14885 | [None](http://www.ncbi.nlm.nih.gov/protein/None) |  |
|  | LQK91_14890 | [None](http://www.ncbi.nlm.nih.gov/protein/None) |  |
|  | LQK91_14895 | [None](http://www.ncbi.nlm.nih.gov/protein/None) |  |
|  | LQK91_14900 | [None](http://www.ncbi.nlm.nih.gov/protein/None) |  |
|  | LQK91_14905 | [None](http://www.ncbi.nlm.nih.gov/protein/None) |  |
|  | LQK91_14910 | [None](http://www.ncbi.nlm.nih.gov/protein/None) |  |
|  | LQK91_14915 | [None](http://www.ncbi.nlm.nih.gov/protein/None) |  |
|  | LQK91_14920 | [None](http://www.ncbi.nlm.nih.gov/protein/None) |  |
